# Bioenhancing effects of piperine and curcumin on triterpenoid pharmacokinetics and neurodegenerative metabolomes from *Centella asiatica* extract in beagle dogs

**DOI:** 10.1038/s41598-022-24935-7

**Published:** 2022-12-01

**Authors:** Tussapon Boonyarattanasoonthorn, Teetat Kongratanapasert, Arnatchai Maiuthed, Robert Hamlin, Anusak Kijtawornrat, Phisit Khemawoot

**Affiliations:** 1 Department of Physiology, Faculty of Veterinary Science, Chulalongkorn University, Pathumwan, Bangkok, 10330 Thailand; 2 Chakri Naruebodindra Medical Institute, Faculty of Medicine Ramathibodi Hospital, Mahidol University, Bang Phli, Samut Prakarn, 10540 Thailand; 3 Department of Pharmacology, Faculty of Pharmacy, Mahidol University, Bangkok, Thailand; 4 Centre of Biopharmaceutical Science for Healthy Ageing, Faculty of Pharmacy, Mahidol University, Bangkok, Thailand; 5 Department of Veterinary Biosciences, College of Veterinary Medicine, The Ohio State University, Columbus, OH USA

**Keywords:** Drug discovery, Medical research

## Abstract

Centell-S is a water-soluble extract of *Centella asiatica* containing more than 80% w/w triterpenoid glycosides. Madecassoside and asiaticoside are two major components of the extract and can be converted into active metabolites, triterpenic acids in large mammal species. In this study, the pharmacokinetic profiles and metabolomic changes generated by the bioactive triterpenoids of Centell-S alone, and in combination with the bioenhancers piperine and curcumin, were investigated in beagle dogs. The test substances were orally administered over multiple doses for 7 consecutive days. At day 1 and 7 after receiving the test compounds, the level of major bioactive triterpenoids and related metabolites were measured using triple quadrupole and high-resolution accurate mass orbitrap models of LCMS to determine pharmacokinetic and metabolomic profiles, respectively. Centell-S was well tolerated, alone and in all combination groups. The combination of Centell-S and piperine significantly increased (*p* < 0.05) the systemic exposure of madecassoside on day 1 and asiatic acid on day 7, by approximately 1.5 to 3.0-fold of C_max_ and AUC values as compared to the Centell-S alone, while the addition of curcumin did not provide a significant improvement. Several metabolomic changes were observed from pre-dose to 4 h post-dose, with some biomarkers of neurodegenerative diseases including l-glutamine, lysophosphatidylcholine (17:0), taurochenodeoxycholic acid, uric acid, stearic acid, palmitic acid, and lactic acid showing good correlation with the systemic exposure of the bioactive triterpenoids (asiatic acid). Thus, the combining of piperine to Centell-S exhibits the improvement of bioactive triterpenoids which are related to the biomarkers of neurodegenerative diseases. These promising results might be useful for the development of this standardised extract to become a more effective phytomedicine for neurodegenerative diseases.

## Introduction

**Figure 1:**
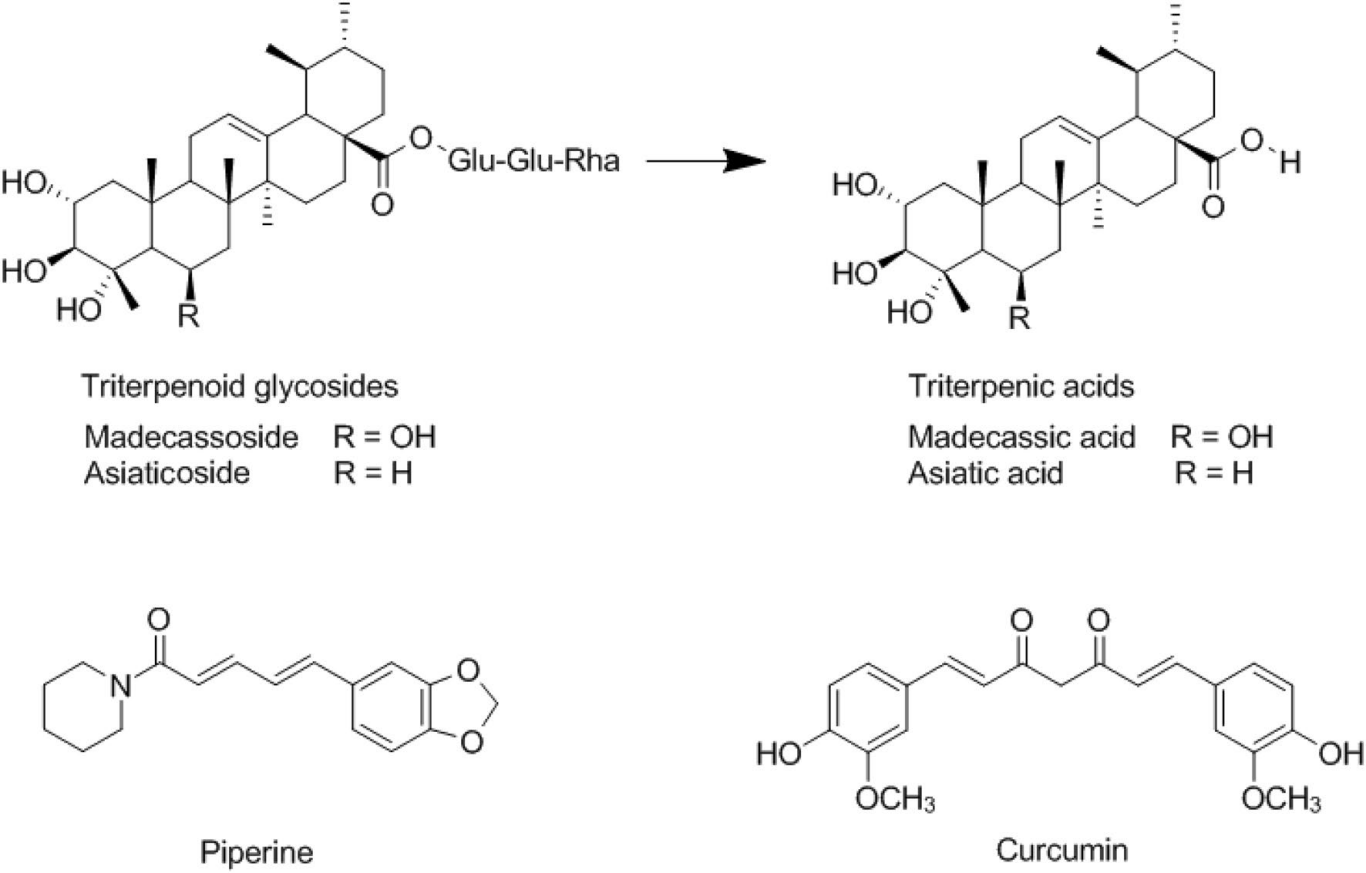
Chemical structures of piperine, curcumin and the bioactive triterpenoids of *Centella asiatica* extract.

*Centella asiatica* is a member of the Umbelliferae family and can be found in tropical areas around the world, such as Southeast Asia, South Africa, Southern America and Europe^1,2^. It is used in traditional medicine for various treatments, such as wound healing, anti-inflammation, anti-bacteria and memory enhancement. *C. asiatica* is mainly composed of triterpenoid glycosides (Fig. 1), of which, madecassoside and asiaticoside are the main components^3–6^. Madecassoside and asiaticoside can be converted into the active metabolites such as asiatic acid by anaerobic bacteria in the gastrointestinal tract of large mammal species^7,8^. Most of the commercial standardised extracts of *C. asiatica* are sparingly water soluble, resulting in low oral bioavailability. Many factors are related to oral bioavailability, including the stability of the drug in the gastrointestinal system, water solubility, membrane permeability and hepatic first-pass metabolism^9,10^. There are several strategies to overcome with these limitations, including physical modification to improve dissolution and permeation, chemical modification of the drug using derivatives, and combination of the drug with bioenhancers to increase oral bioavailability and bioefficacy^11,12^.

A bioenhancer is an agent that is co-administered with a drug to enhance bioavailability and efficacy, while having minimal pharmacological effect on the drug^13^. The expected outcomes from the addition of bioenhancers are an improvement of drug absorption and an increase in drug concentration in the blood and/or tissues. Natural compounds with bioenhancing properties such as piperine, curcumin, quercetin and glycyrrhizin, are used with traditional and modern medicines. Piperine is the world’s first alkaloid purified bioenhancer molecule and is extracted from *Piper longum*, whose mechanism of action involves the increase of drug absorption in the gastrointestinal system or the inhibition of drug metabolising enzymes, such as cytochrome P450, which contribute to the hepatic first-pass metabolism of many drugs^14^. In some studies, piperine increased the absorption of drugs via the gastrointestinal system by vasodilation and the increased permeability of the cell membrane to transport more drug molecules^15^. Curcumin is the major curcuminoid extracted from the rhizome of *Curcuma longa* and exhibits bioenhancing effects. Its mechanism of action is the modulation of the activity of both specific and non-specific drug metabolising enzymes in the liver and the inhibition of drug transporter P-glycoprotein^16,17^. Different studies using curcumin as a bioenhancer have shown inconsistent results, demonstrating both a reduction and an enhancement of drug absorption^18^. Several studies using curcumin as a bioenhancer combined with anticancer drugs or antimicrobial agents have demonstrated the increased bioavailability of these agents, in one study the bioavailability of docetaxel in combination with 100 mg/kg of curcumin increased approximately eight-fold compared to the control group^19^, while pre-treatment with curcumin 60 mg/kg increased the AUC of norfloxacin approximately two-fold compared with the control group^20^.

*C. asiatica* has long been used in traditional medicine and its active components exhibit a wide range of neuroprotective and cognitive benefits^21,22^. In many studies, *C. asiatica* extract exhibits the cognitive enhancing effects in normal animals as well as in neurodegenerative disease models^23–25^. Kumar et al. found the improvement of memory and reduction of the mitochondrial dysfunction in the aging mouse model after receiving the *C. asiatica* extract^26^. In other studies, *C. asiatica* exhibits protective effects against hippocampal dysfunction which plays an important role in learning and memory as studied in the Alzheimer's disease model^27^. Neurodegenerative disease such as Alzheimer's disease, Parkinson's disease, and Huntington's disease are known to cause progressive and irreversible memory loss, development of bradykinesia, and progressive dementia due to the impairment of metabolisms^28^. These clinical expressions are delayed and exhibit severe phases of disease progression. To overcome this limitation, presymptomatic diagnosis using metabolomics could be a potential method^29^. Because metabolomic changes by biochemical reactions in vivo could refer to progression of neurodegenerative diseases^30^.

Given that the use of standardised extracts of *C. asiatica* is increasing, and their bioavailability is low, it is of interest to find effective bioenhancers to improve the pharmacological activities of these standardised extracts. Therefore, this study aimed to investigate the bioenhancing effects of piperine and curcumin on the bioactive triterpenoids of *C. asiatica* in beagle dogs. Additionally, the metabolomic profiles were observed to identify the metabolomes related to neurodegenerative diseases. The dose selection of the standardised extract of *C. asiatica* (Centell-S) was based on our previous study and on the United States Food and Drug Administration (USFDA) guidance for industry on dose selection among mammals^7,31–33^. The results obtained from this study will be of benefit in understanding the pharmacokinetic profiles and metabolomics changes for the development of standardised extracts of Centell-S in the near future.

## Results

### Animal tolerability

Following oral administration of the test substances, all dogs showed normal physical appearance and behaviour, with no adverse events or clinical signs of behavioural changes observed between 0–24 h after dosing. The mean biochemical profiles obtained following administration of the test substances were in the normal range and similar to baseline values in all experimental groups (Table 1). The test substances in this study were well tolerated by all dogs.

**Table 1:** Physical and biochemical profiles of experimental beagles.

Biochemical parameters	Experimental groups								
	Baseline	CTS 20 mg/kg p.o. Day 1	CTS 20 mg/kg p.o. Day 7	CTS + Pip 20 mg/kg p.o. Day 1	CTS + Pip 20 mg/kg p.o. Day 7	CTS + Cur 20 mg/kg p.o. Day 1	CTS + Cur 20 mg/kg p.o. Day 7	CTS + Pip + Cur 20 mg/kg p.o. Day 1	CTS + Pip + Cur 20 mg/kg p.o. Day 7
Physical appearance	Normal	Normal	Normal	Normal	Normal	Normal	Normal	Normal	Normal
Body weight	13.95 ± 1.36	14.24 ± 1.52	14.35 ± 1.43	14.11 ± 1.39	14.52 ± 1.18	14.08 ± 1.60	14.41 ± 1.59	14.39 ± 1.43	14.61 ± 1.25
White blood cell (× 10^3^/µL)	10.30 ± 1.40	10.95 ± 1.83	11.13 ± 0.73	10.18 ± 1.64	11.13 ± 1.31	10.55 ± 1.64	10.45 ± 1.65	10.25 ± 1.59	10.35 ± 1.72
Red blood cell (× 10^6^/µL)	7.08 ± 0.28	6.64 ± 0.13	7.02 ± 0.19	7.10 ± 0.37	7.18 ± 0.89	6.26 ± 0.23	6.14 ± 0.25	6.86 ± 0.72	6.90 ± 0.62
Hemoglobin (g/dL)	16.4 ± 0.7	15.5 ± 0.3	16.1 ± 0.3	16.0 ± 0.6	15.98 ± 1.7	14.8 ± 0.4	14.2 ± 0.4	15.6 ± 1.4	15.3 ± 0.7
Platelet (× 10^3^/µL)	251 ± 37	255 ± 32	254 ± 55	237 ± 62	235 ± 72	255 ± 41	232 ± 40	258 ± 39	266 ± 50
Blood urea nitrogen (mg/dL)	11.4 ± 0.5	11.6 ± 1.7	10.9 ± 0.8	12.3 ± 1.1	11.8 ± 2.0	11.4 ± 2.0	11.4 ± 1.2	12.3 ± 1.8	12.1 ± 1.9
Creatinine (mg/dL)	0.6 ± 0.1	0.6 ± 0.1	0.6 ± 0.1	0.8 ± 0.1	0.7 ± 0.2	0.6 ± 0.1	0.6 ± 0.1	0.8 ± 0.2	0.7 ± 0.2
AST (U/L)	27 ± 5	25 ± 5	27 ± 3	24 ± 4	22 ± 4	24 ± 5	23 ± 2	23 ± 5	24 ± 4
ALT (U/L)	52 ± 7	49 ± 5	50 ± 11	46 ± 10	45 ± 9	45 ± 9	40 ± 6	41 ± 4	41 ± 4
Alkaline phosphatase (U/L)	76 ± 31	72 ± 29	84 ± 36	63 ± 40	68 ± 35	81 ± 36	74 ± 29	64 ± 32	63 ± 27

*AST* aspartate transaminase, *ALT* alanine transaminase, *CTS* Centell-S, *Pip* piperine, *Cur* curcumin. Data are expressed as mean ± SD, (n = 4); *p < 0.05 for significant differences. Decimal numbers were reported according to laboratory standard of Small Animal Hospital, Faculty of Veterinary Science, Chulalongkorn University.

### Plasma concentration–time profiles

**Figure 2:**
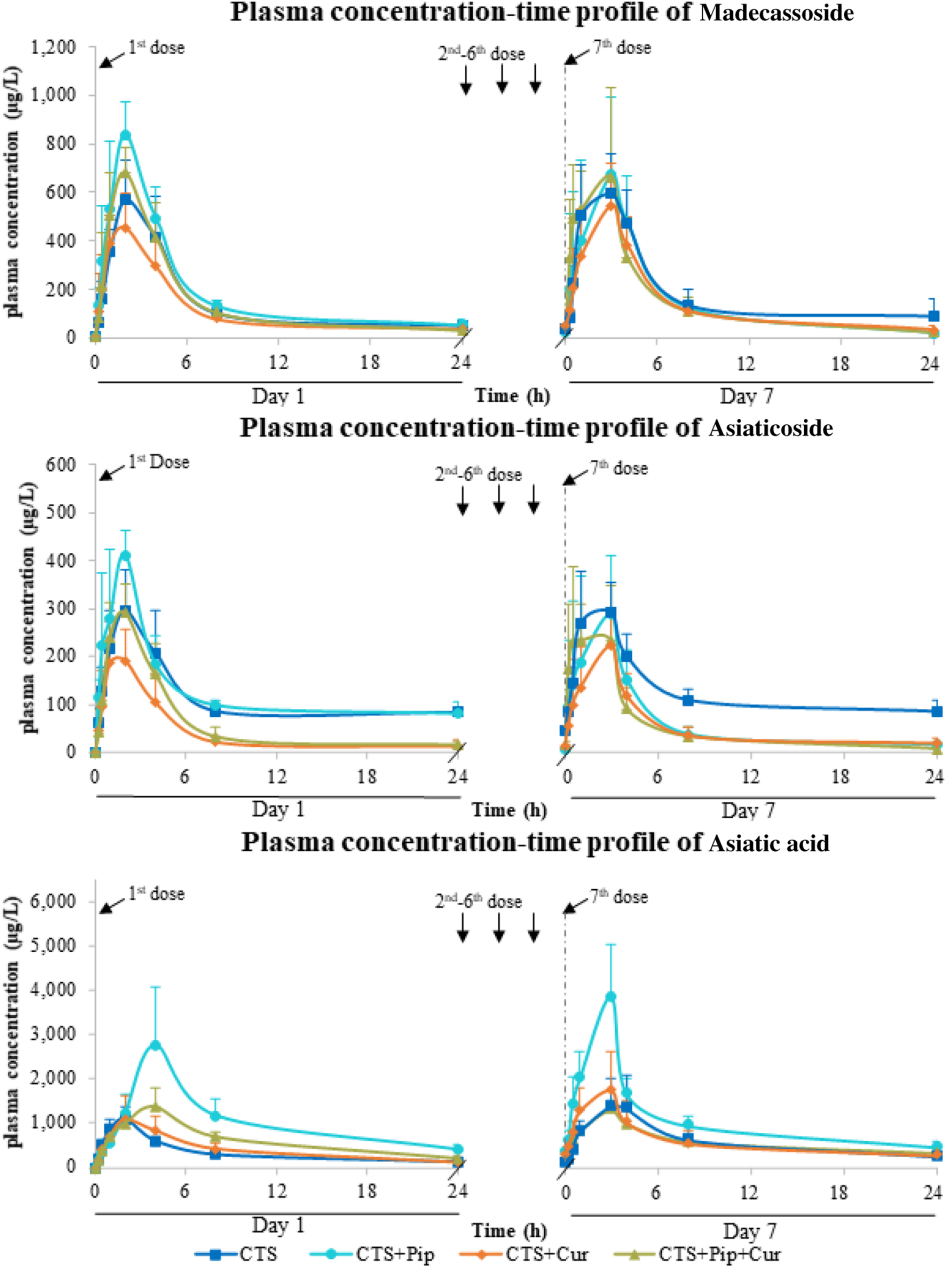
Plasma concentration–time profiles of major bioactive triterpenoids from *Centella asiatica* extract alone and in combination with curcumin and/or piperine.

The major bioactive triterpenoids found in the plasma after oral administration of Centell-S 20 mg/kg were madecassoside, asiaticoside and asiatic acid (Fig. 2). The two parent triterpenoid glycosides, madecassoside and asiaticoside, both reached maximum plasma concentration within 1–2 h of oral dosing on day 1, and then showed a slight delay (2–4 h) on day 7 in most tested groups. The major active metabolite, asiatic acid, showed a maximum concentration at 2–4 h after oral administration of Centell-S on day 1, which was slightly slower than the parent triterpenoid glycosides. Interestingly, the maximum level of asiatic acid was 2–5-fold higher than madecassoside and asiaticoside in all test groups. Asiatic acid also showed a tendency to accumulate after continuous dosing for 7 days in all test groups, with an accumulation ratio of 1.05–1.44. The combination of Centell-S and piperine increased the plasma level of madecassoside on day 1, and asiatic acid on both day 1 and day 7 by approximately 1.5–3.0-fold. On the contrary, the combination of Centell-S and curcumin showed slightly decreased plasma concentration levels of madecassoside and asiaticoside on day 1. The triple combination of Centell-S plus piperine and curcumin did not produce significant changes in the plasma concentration of any of the bioactive triterpenoids compared with Centell-S alone on either day 1 or day 7.

### Pharmacokinetic parameters

The non-compartmental pharmacokinetic parameters of the major bioactive triterpenoids are summarised in Tables 2 and 3. Among the experimental groups that received different bioenhancers, the C_max_ values for madecassoside (875.95 ± 101.63 µg/L), asiaticoside (411.53 ± 13.15 µg/L) and asiatic acid (3751.53 ± 1219.25 µg/L) were the highest in the group that received oral administration of Centell-S plus piperine, and were significantly higher than the group that received Centell-S alone (*p* < 0.05). On the other hand, the C_max_ value of asiaticoside (217.38 ± 61.62 µg/L) after oral administration of Centell-S plus curcumin exhibited a significantly lower value than the group that received an oral administration of Centell-S alone (*p* < 0.05). The AUC value of both triterpenoid glycosides and triterpenic acid showed a similar tendency to the C_max_ values: combination with piperine increased the AUC, while combination with curcumin was likely to decrease the AUC. Excretion of the triterpenoid glycosides after oral administration was mainly via the faecal route, which occurred within 24 h of administration. For the active metabolites, triterpenic acids were found in negligible amounts in the faeces of all experimental groups (Table 4).

**Table 2:** Pharmacokinetic parameters of madecassoside after oral administration of 20 mg/kg Centell-S, 20 mg/kg Centell-S + 20 mg/kg piperine, 20 mg/kg Centell-S + 20 mg/kg curcumin, and 20 mg/kg Centell-S + 20 mg/kg piperine + 20 mg/kg curcumin as repeated doses.

Pharmacokinetic parameters	Experimental groups							
	CTS 20 mg/kg p.o. Day 1	CTS 20 mg/kg p.o. Day 7	CTS + Pip 20 mg/kg p.o. Day 1	CTS + Pip 20 mg/kg p.o. Day 7	CTS + Cur 20 mg/kg p.o. Day 1	CTS + Cur 20 mg/kg p.o. Day 7	CTS + Pip + Cur 20 mg/kg p.o. Day 1	CTS + Pip + Cur 20 mg/kg p.o. Day 7
	**(a) Parent compounds (Madecassoside)**							
C_max_^a^ (µg/L)	503.77 ± 120.71	681.33 ± 135.60	875.95 ± 101.63*	818.83 ± 222.25	492.75 ± 98.94	573.08 ± 158.92	693.35 ± 86.05	759.83 ± 324.56
T_max_^b^ (h)	2.00 (0.00)	1.50 (1.00)	2.00 (0.25)	3.00 (2.00)	2.00 (0.50)	3.00 (2.00)	1.50 (1.00)	1.00 (0.63)
AUC_0–24_^a^ (µg h/L)	3798 ± 837	4734 ± 347	4958 ± 229*	4891 ± 1080	3173 ± 543	3771 ± 322	3963 ± 363	4647 ± 757
AUC_0–inf_^a^ (µg h/L)	4300 ± 727	5044 ± 214	5367 ± 420*	5164 ± 1215	3766 ± 1533	3903 ± 190	4480 ± 557	4970 ± 613
Rate of bioavailability (1/h)	1.07 ± 0.41	2.31 ± 1.46	1.39 ± 1.19	0.83 ± 0.61	1.92 ± 1.09	1.17 ± 0.55	1.49 ± 0.88	1.55 ± 1.07
MRT^a^ (h)	23.57 ± 7.56	25.10 ± 12.02	21.23 ± 6.19	10.60 ± 4.94	20.83 ± 17.41	6.77 ± 3.85	14.80 ± 3.31	11.50 ± 3.72
Vd^a^ (L/kg)	44.05 ± 14.84	45.10 ± 25.26	25.40 ± 5.97	18.88 ± 3.60	23.90 ± 8.49	23.93 ± 2.55	19.80 ± 5.96	17.10 ± 7.81
CL^a^ (L/h/kg)	1.94 ± 0.59	1.13 ± 0.80	1.59 ± 0.28	1.85 ± 0.42	1.98 ± 0.53	2.03 ± 0.68	2.08 ± 0.27	1.88 ± 0.26
Half-life^b^ (h)	13.33 (5.94)	11.61 (6.87)	8.45 (2.63)	6.46 (2.99)	7.27 (0.20)	7.18 (0.63)	5.30 (2.22)	5.27 (1.70)

^a^Data are expressed as mean ± SD; ^b^Data are expressed as median (IQR); *p < 0.05 for significant differences from the CTS group. *C*_*max*_ maximum plasma concentration, *T*_*max*_ time to reach C_max_, *AUC*_*0–24*_ area under the plasma concentration–time curve from time 0–24 h, *AUC*_*0–inf*_ area under the plasma concentration–time curve from time 0–infinity, *MRT* mean resident time, *Vd* volume of distribution, *CL* clearance, *CTS* Centell-S, *Pip* piperine, *Cur* curcumin.

**Table 3:** Pharmacokinetic parameters of asiaticoside (a) and asiatic acid (b) after oral administration of 20 mg/kg Centell-S, 20 mg/kg Centell-S + 20 mg/kg piperine, 20 mg/kg Centell-S + 20 mg/kg curcumin, and 20 mg/kg Centell-S + 20 mg/kg piperine + 20 mg/kg curcumin as repeated doses.

Pharmacokinetic parameters	(a) Experimental groups							
	CTS 20 mg/kg p.o. Day 1	CTS 20 mg/kg p.o. Day 7	CTS + Pip 20 mg/kg p.o. Day 1	CTS + Pip 20 mg/kg p.o. Day 7	CTS + Cur 20 mg/kg p.o. Day 1	CTS + Cur 20 mg/kg p.o. Day 7	CTS + Pip + Cur 20 mg/kg p.o. Day 1	CTS + Pip + Cur 20 mg/kg p.o. Day 7
	**(a) Parent compounds (Asiaticoside)**							
C_max_^a^ (µg/L)	339.17 ± 58.20	354.23 ± 69.57	441.53 ± 13.15	380.10 ± 72.27	217.38 ± 61.62*	226.13 ± 63.89*	314.45 ± 38.90	376.03 ± 23.99
T_max_^b^ (h)	2.00 (0.00)	1.50 (1.00)	2.00 (0.25)	2.00 (0.50)	1.50 (1.50)	2.00 (0.00)	1.50 (1.00)	0.75 (0.75)
AUC_0–24_^a^ (µg h/L)	2844 ± 315	3364 ± 449	3146 ± 288	1812 ± 500*	1245 ± 429*	1379 ± 224*	1638 ± 263*	1536 ± 180*
AUC_0–inf_^a^ (µg h/L)	5664 ± 385	5997 ± 458	5687 ± 428	2144 ± 623*	1452 ± 613*	1723 ± 552*	1941 ± 315*	1599 ± 147*
Rate of bioavailability (1/h)	1.29 ± 0.56	2.65 ± 2.05	1.32 ± 0.81	0.90 ± 0.58	1.80 ± 0.38	1.10 ± 0.52	1.70 ± 1.06	1.56 ± 0.70
MRT^a^ (h)	63.30 ± 11.60	58.33 ± 18.58	61.87 ± 34.24	22.58 ± 8.92	19.20 ± 11.02	33.30 ± 13.98	27.30 ± 5.11	19.33 ± 3.74
Vd^a^ (L/kg)	81.70 ± 5.20	62.60 ± 14.01	63.45 ± 10.99	74.73 ± 25.05	98.90 ± 21.70	107.53 ± 42.64	109.35 ± 52.20	107.10 ± 7.81
CL^a^ (L/h/kg)	0.58 ± 0.25	0.94 ± 0.52	1.02 ± 0.52	2.02 ± 1.02	4.27 ± 3.88	4.07 ± 1.46	4.06 ± 0.78	4.21 ± 0.39
Half-life^b^ (h)	19.55 (6.20)	23.13 (14.37)	19.35 (15.64)	19.68 (2.79)	20.79 (19.63)	17.32 (5.92)	13.83 (5.75)	9.45 (2.53)
	**(b) Active metabolites (asiatic acid)**							
C_max_^a^ (µg/L)	1210.83 ± 52.13	1499.10 ± 702.92	3207.93 ± 1164.85*	3751.53 ± 1219.25*	1110.03 ± 506.20	1369.53 ± 403.23	1194.53 ± 289.46	1425.45 ± 53.42
T_max_^b^ (h)	2.00 (0.25)	3.00 (2.00)	4.00 (0.00)	2.00 (0.00)	2.00 (0.50)	2.00 (0.00)	4.00 (0.00)	2.00 (0.50)
AUC_0–24_^a^ (µg h/L)	8281 ± 560	11,912 ± 3987	22,803 ± 8253*	25,274 ± 7230*	9847 ± 2971	11,532 ± 4237	14,045 ± 1985*	14,812 ± 2979
AUC_0–inf_^a^ (µg h/L)	10,595 ± 1078	14,483 ± 3711	28,311 ± 8028*	31,926 ± 7191*	11,297 ± 2720	13,147 ± 3718	17,328 ± 1178*	18,346 ± 1794
Rate of bioavailability (1/h)	1.06 ± 0.26	1.02 ± 0.30	0.78 ± 0.13	1.18 ± 0.39	1.33 ± 0.49	1.21 ± 0.12	0.94 ± 0.33	1.22 ± 0.35

^a^Data are expressed as mean ± SD; ^b^Data are expressed as median (IQR); *p < 0.05 for significant differences from the CTS group. *C*_*max*_ maximum plasma concentration, *T*_*max*_ time to reach C_max_, *AUC*_*0–24*_ area under the plasma concentration–time curve from time 0–24 h, *AUC*_*0–inf*_ area under the plasma concentration–time curve from time 0–infinity, *MRT* mean resident time, *Vd* volume of distribution, *CL* clearance, *CTS* Centell-S, *Pip* piperine, *Cur* curcumin.

**Table 4:** Percentage recovery of madecassoside, asiaticoside, madecassic acid, asiatic acid in excreta of all experimental groups.

Percent recovery	Experimental groups							
	CTS 20 mg/kg p.o. Day 1	CTS 20 mg/kg p.o. Day 7	CTS + Pip 20 mg/kg p.o. Day 1	CTS + Pip 20 mg/kg p.o. Day 7	CTS + Cur 20 mg/kg p.o. Day 1	CTS + Cur 20 mg/kg p.o. Day 7	CTS + Pip + Cur 20 mg/kg p.o. Day 1	CTS + Pip + Cur 20 mg/kg p.o. Day 7
	0–24 h	0–24 h	0–24 h	0–24 h	0–24 h	0–24 h	0–24 h	0–24 h
**Urine**								
Madecassoside	7.19 ± 2.94	11.13 ± 0.74	14.85 ± 10.53	11.08 ± 4.62	4.97 ± 1.88	6.08 ± 3.19	5.87 ± 2.17	4.68 ± 2.01
Asiaticoside	2.90 ± 1.85	5.35 ± 1.13	7.29 ± 7.96	6.04 ± 1.68	1.90 ± 0.74	3.11 ± 1.02	3.10 ± 1.28	2.17 ± 0.99
Madecassic acid	< 0.01	< 0.01	< 0.01	< 0.01	< 0.01	< 0.01	< 0.01	< 0.01
Asiatic acid	< 0.01	< 0.01	< 0.01	< 0.01	< 0.01	< 0.01	< 0.01	< 0.01
**Faeces**								
Madecassoside	90.11 ± 12.54	73.13 ± 24.61	86.03 ± 7.99	71.49 ± 39.13	94.72 ± 20.73	95.04 ± 27.87	90.87 ± 18.50	87.44 ± 47.34
Asiaticoside	96.83 ± 12.20	70.43 ± 23.82	93.78 ± 7.60	93.82 ± 58.80	97.30 ± 33.83	90.81 ± 40.45	96.64 ± 15.44	94.41 ± 42.80
Madecassic acid	3.87 ± 1.34	3.56 ± 2.05	3.90 ± 2.81	4.10 ± 4.21	5.02 ± 2.43	6.66 ± 0.85	2.31 ± 0.28	8.92 ± 0.56
Asiatic acid	2.35 ± 0.11	3.10 ± 1.50	4.30 ± 2.08	3.52 ± 3.89	7.34 ± 1.47	8.08 ± 5.18	3.91 ± 1.88	3.60 ± 5.45

*CTS* Centell-S, *Pip* piperine, *Cur* curcumin. Data are expressed as mean ± SD (n = 4).

### Metabolomic profiles

**Figure 3:**
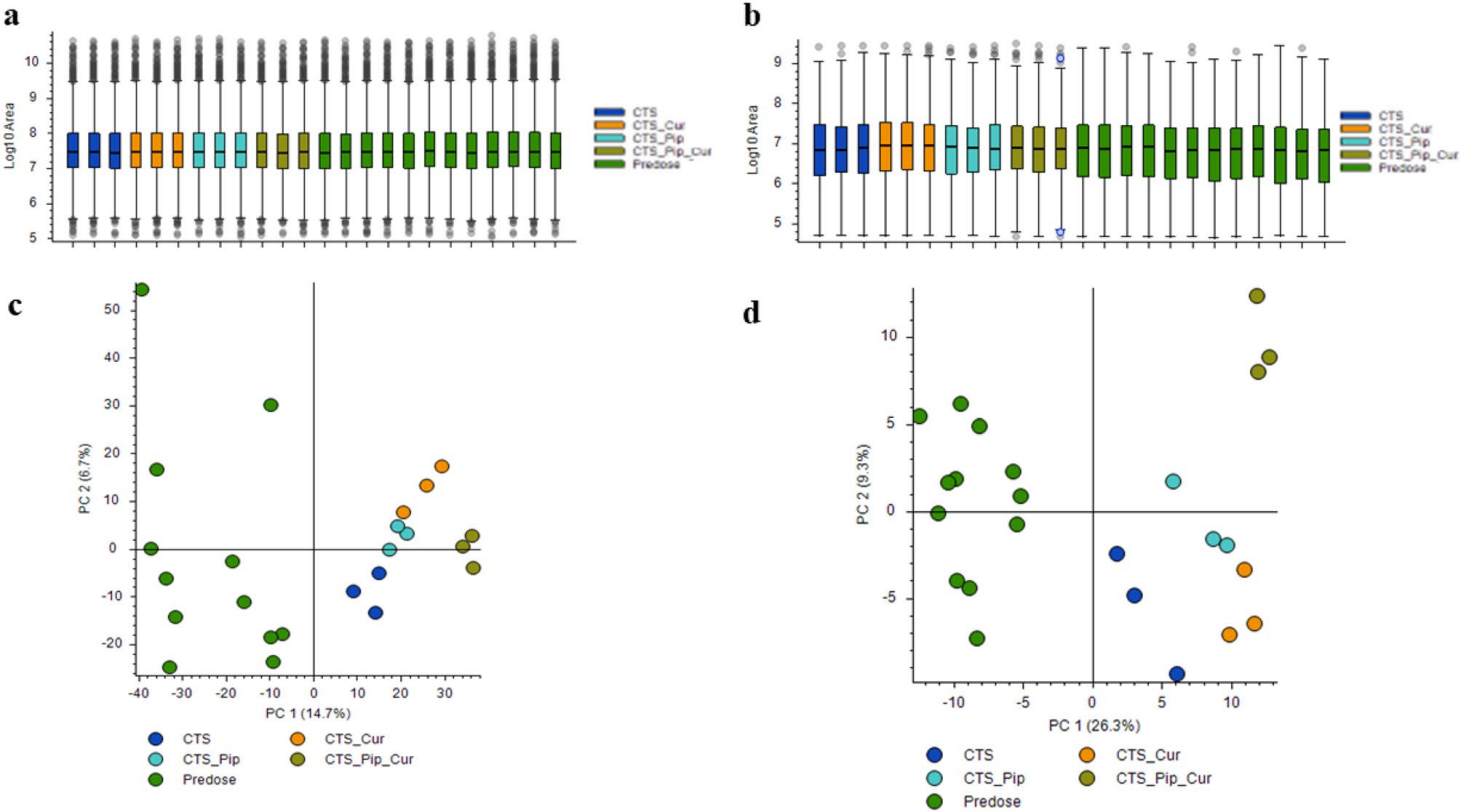
The distribution of mass area in each plasma sample group: *Centella asiatica* (CTS) extract alone, in combination with curcumin (CTS_Cur), in combination with piperine (CTS_Pip) and in combination with both curcumin and piperine (CTS_Pip_Cur) in (**a**) positive mode and (**b**) negative mode. The PCA plot of metabolites from (**c**) positive mode and (**d**) negative mode of each plasma sample group.

**Figure 4:**
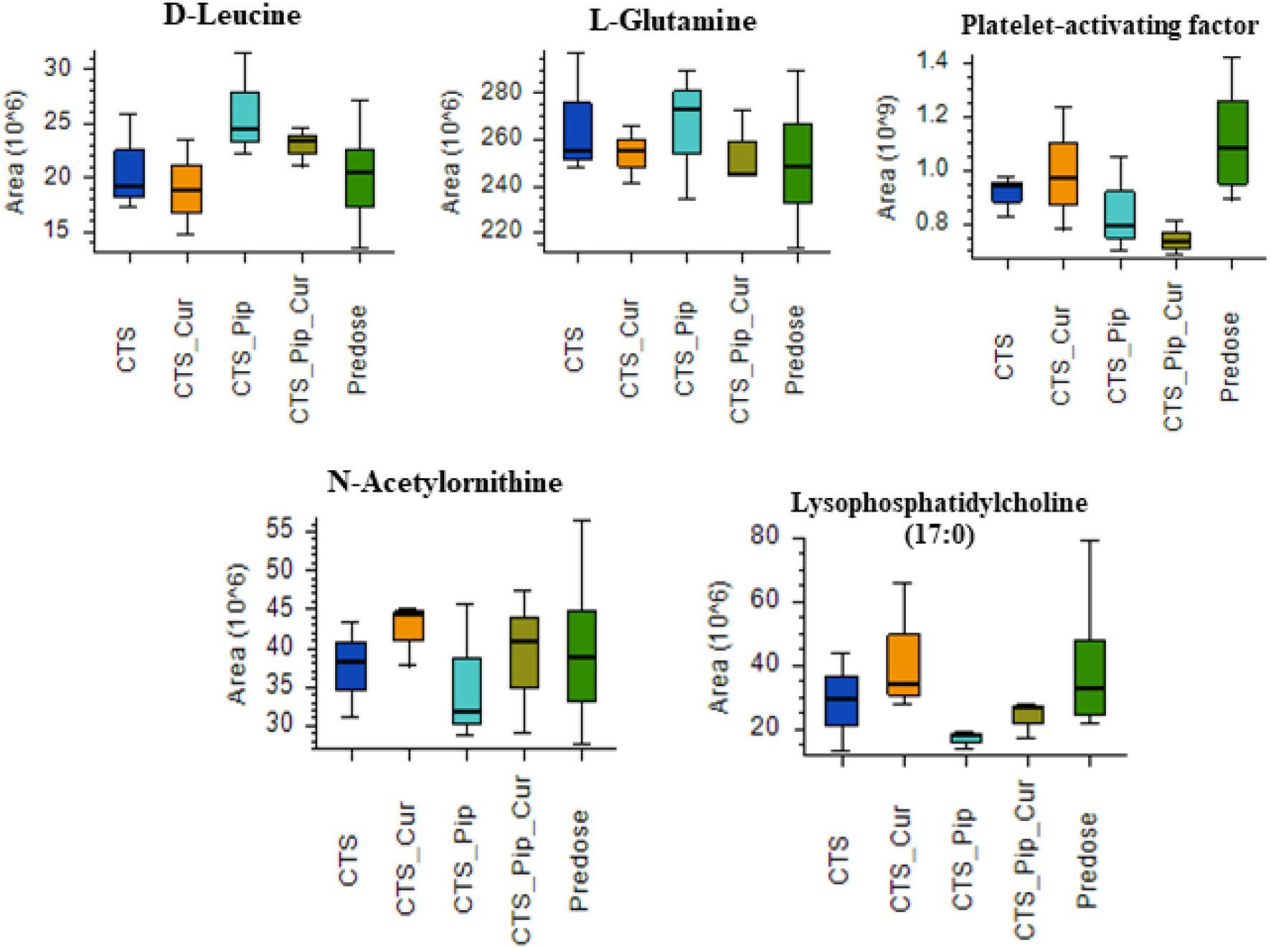
Boxplots of the metabolite biomarkers that changed 4 h after administration of *Centella asiatica* (CTS) extract alone, in combination with curcumin (CTS_Cur), in combination with piperine (CTS_Pip) and in combination with both curcumin and piperine (CTS_Pip_Cur) in the positive mode of detection.

**Figure 5:**
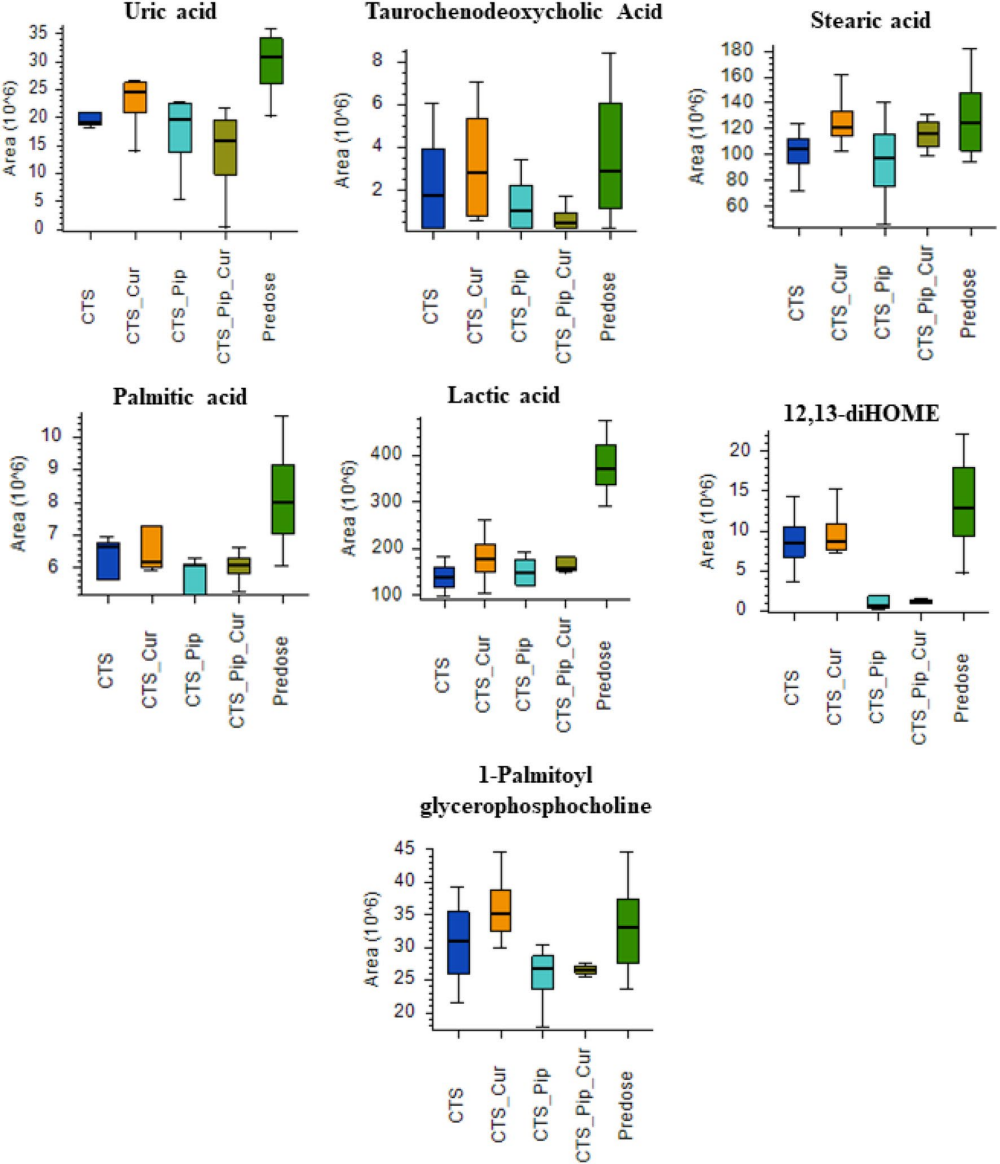
Boxplots of the metabolite biomarkers that changed 4 h after administration of *Centella asiatica* (CTS) extract alone, in combination with curcumin (CTS_Cur), in combination with piperine (CTS_Pip) and in combination with both curcumin and piperine (CTS_Pip_Cur) in the negative mode of detection.

The metabolite components of the plasma extracts at pre-dose and 4 h post-dose of all intervention groups Centell-S (CTS) alone, combination with curcumin (CTS_Cur), combination with piperine (CTS_Pip) and combination with both curcumin and piperine (CTS_Pip_Cur) were separated by UHPLC. The mass to charge ratios were then determined by tandem mass spectrometer (Q-Orbitrap), the compounds were then identified using many databases, with the aid of Compound Discoverer Software, as indicated in the “Materials and methods” section. A total of 4680 metabolites were identified in positive ion mode and 595 metabolites were identified in negative ion mode (data not shown). The metabolite area distribution of the plasma samples identified the similar pattern of distribution between samples (Fig. 3a,b). Principal component analysis (PCA) was used to indicate whether each intervention altered the plasma metabolite profiles (Fig. 3c,d), with results indicating the separated group of plasma metabolite profiles that resulted from different interventions, in both positive and negative detection mode. To evaluate the effect of the active compound, asiatic acid, on the change in some metabolites in the plasma, we selected metabolites whose altered levels correlated with the level of asiatic acid and whose function was exhibited according to the asiatic acid pharmacological effect, especially with regard to the neurological aspect^34–38^. In the positive detection mode, we found 5 metabolites, d-leucine, l-glutamine, platelet-activating factor, *N*-acetylornithine and lysophosphatidylcholine (17:0), as shown in Fig. 4. In the negative detection mode, we found 7 metabolites: uric acid, taurochenodeoxycholic acid, stearic acid, palmitic acid, lactic acid, 12,13-dihydroxy-9Z-octadecenoic acid (12,13-diHOME) and 1-palmitolyglycerophosphocholine (Fig. 5).

## Discussion

*C. asiatica* extract, which contains bioactive triterpenoid glycoside components, has been used in traditional medicine for many centuries and has a wide range of medicinal values, with minimal toxicity. In this study, the standardised extract of *C. asiatica* (Centell-S) was administered alone and in combination with piperine and/or curcumin as bioenhancers. The test substances were well tolerated during the study, and dogs in all experimental groups showed no signs of toxicity and no changes in physical appearance. Biochemical parameters indicating the health status, liver and kidney function of the animals were stable and within the normal range pre- and post-administration of the test substances, indicating that the test compound has a good safety profile in animals, in line with many pre-clinical and clinical studies of *C. asiatica*^39–43^. Most commercial standardised extracts of *C. asiatica* have low oral bioavailability due to poor water solubility, and this problem was minimised by the development of a water-soluble extract of *C. asiatica* (Centell-S)^39,44^. Centell-S is freely soluble in water, with the absolute oral bioavailability improved approximately twofold, as described in our previous study^7^. However, the development of this phytopharmaceutical product for the improvement of systemic exposure and metabolomic changes in experimental animals is still ongoing.

In this study, the bioenhancers piperine and curcumin were administered to enhance the pharmacokinetic and metabolomic profile of Centell-S. The plasma concentration–time profiles of madecassoside and asiatic acid increased approximately 1.5–3.0-fold after oral administration of Centell-S with piperine, compared to Centell-S alone. Coadministration with piperine also significantly enhanced the C_max_ and AUC of madecassoside and asiatic acid, which implies that piperine could improve the systemic exposure of triterpenoid glycosides and the active metabolite, asiatic acid. These findings correlate with other reports; for example, Islam et al*.* reported that phytosomes with piperine inhibit P-glycoprotein, thus significantly increasing the oral bioavailability of domperidone in comparison with the pure drug suspension^45^. Izgelov et al*.* also reported that the use of piperine in combination with cannabidiol in self-emulsifying drug delivery systems could improve the oral bioavailability of cannabidiol approximately 2.5-fold compared to the control group^46^, and a study by Bansal et al*.* reported that the coadministration of irinotecan and piperine exhibited a reduction in the Vd of the drug via P-glycoprotein inhibition^47^. It is possible that the increased systemic exposure of bioactive triterpenoids from Centell-S might be related to P-glycoprotein inhibition.

Asiaticoside is biotransformed to asiatic acid by beta-glycosidases that are released from anaerobic bacteria in the intestine^48–50^. In our study, the highest biotransformation of asiaticoside to asiatic acid was found in the group that received Centell-S plus piperine, while the group that received a coadministration with curcumin exhibited a decrease in the plasma concentration of asiatic acid compared to the group receiving Centell-S alone. This implies that curcumin has the potential to inhibit the biotransformation processes of asiaticoside to asiatic acid in the gut lumen. The coadministration of Centell-S with curcumin did not provide a significant improvement in the C_max_ or AUC of madecassoside or asiatic acid, which is consistent with a study by Volak et al*.*, who reported that the use of curcumin is unlikely to result in a clinically significant interaction involving drug metabolising enzymes^51^. The excretion of madecassoside and asiaticoside occurs via faeces, which is the major route of excretion. The active metabolite, asiatic acid, was also found in faeces within 24 h of administration of the test substances in all groups, indicating that gut microbiota could metabolise the parent compound to the active metabolite, in line with the human study reported by Songvut et al*.*^8^.

Previous studies have indicated that the bioactive components of *C. asiatica* exert properties to improve brain function or prevent some neurodegenerative disorders. Hongqun Ding et al*.* reported that asiatic acid exhibits antioxidant property which inhibit apoptosis inducing by translocation of α-synuclein into mitochondria^52^, while Dong Chen et al*.* indicated that antioxidant effect of asiatic acid protected dopaminergic neuron from neuroinflammation^53^. As neurological diseases can alter biochemical substances in the brain, which can be observed as changes in related metabolites in plasma^54^, a metabolomic approach was implemented to investigate changes in the plasma of dogs, 4 h after receiving Centell-S alone and in combination with bioenhancers. Neurological disease-related metabolites that changed along with the plasma level of the active metabolite, asiatic acid, were elucidated by both a positive and negative mode of detection. In the positive mode of detection, we found 5 metabolites that changed in a similar way to the plasma asiatic acid but 4 metabolites have a role that is related to neurodegenerative disorders. These 4 metabolites were l-glutamine, platelet-activating factor, *N*-acetylornithine and lysophosphatidylcholine (17:0). The amino acid that correlated with the plasma asiatic acid was l-glutamine. Jianmin et al*.* suggested that higher levels of both intracellular and extracellular l-glutamine provided a neuroprotective effect against β-amyloid and hydrogen peroxide induced neuronal DNA damage^55^. The other metabolites from the positive mode of detection, platelet-activating factor, *N*-acetylornithine and lysophosphatidylcholine (17:0), act as biomarkers of neurological diseases and were found to have an inverted correlation with the plasma concentration of asiatic acid. Platelet-activating factor is an inflammatory phospholipid signalling molecule that can promote immune-mediated neuronal inflammation and alter synaptic plasticity^56^, reduce learning and memory capacity^57–59^, and induce neurotoxicity^60,61^. A study by Weng et al. indicated that Alzheimer’s patients with mild cognitive impairment have higher levels of acetylornithine than those seen in normal aging^62^, and lysophosphatidylcholine has been found to be a negative mediator of neuronal integrity, mediating pericyte loss and vascular barrier disruption^63,64^, down regulating myelination and triggering a defect of the motor neuron function^65^. Moreover, lysophosphatidylcholine promoted the neurotoxicity of β1–42 peptide by enhancing oligomer formation and neurotoxic protein aggregation^66^.

In the negative mode of detection, we found 7 metabolites that inversely changed with plasma asiatic acid: uric acid, taurochenodeoxycholic acid, stearic acid, palmitic acid, lactic acid, 12,13-diHOME and 1-palmitolyglycerophosphocholine. These metabolites are found at higher levels when neurological conditions worsen. Previous studies have indicated that high serum levels of uric acid are associated with the progression of neurological diseases: a preclinical study showed that serum uric acid may promote hippocampal inflammation, which may induce cognitive dysfunction^67^, and clinical studies have indicated that elderly people with elevated levels of serum uric acid may have a higher risk of vascular or mixed dementia^68^ and that these levels may increase cerebral ischemic pathology^69^, which is associated with lower neuropsychological assessment scores, particularly in word fluency tests and number connection tests^70^. Taurochenodeoxycholic acid belongs to the bile acid family and can be found in small amounts in the circulation in healthy individuals, but can be elevated in certain health conditions, such as liver disease and aging^71,72^. Bile acids, including taurochenodeoxycholic acid, have been shown to cross the blood brain barrier, contributing to a neurological decline through the activation of the nuclear receptor farnesoid X receptor^73,74^. In the case of saturated fatty acids, stearic acid and palmitic acid were found to correlate with the plasma concentration of the active compound. A study by Heude et al. indicated that higher proportions of stearic acid (saturated fatty acid, 18:0) and N6 polyunsaturated fatty acids promoted the risk of cognitive impairment^75^, while previous studies have also indicated that high levels of palmitic acid are found in the frontal cortex of patients with Parkinson’s disease and the parietal cortex of Alzheimer’s patients, which are associated with the neurodegenerative stage of these diseases^76,77^. In animal models, palmitic acid induces hippocampal inflammation and promotes the release of TNF-alpha, which inhibits synaptic plasticity and triggers memory impairment^78^. Lactic acid is one of the markers of an alteration in metabolism in the body, with several reports showing elevated levels of lactic acid in the cerebrospinal fluid of aging humans^79,80^, while an animal model using 1H-NMR detection confirmed that lactic acid levels in 88- to 96-week-old rats were significantly increased^81^. Moreover, high lactic acid concentrations in the brain could predict brain aging^82^. Oxidised linoleic acid and 12,13-diHOME levels were associated with low performance in tests of psychomotor processing speed, attention and executive function^83^. Finally, a study of age-matched controls with Alzheimer’s disease indicated that all choline metabolites in cerebrospinal fluid were significantly increased in Alzheimer’s disease, especially glycerophosphocholine, which might be mediated by phospholipase A2^84^. Further efficacy studies of Centell-S in animal models of neurodegenerative disease are urgently required to elucidate the pharmacological activity for phytopharmaceutical product development.

## Conclusion

The combining of piperine as a bioenhancer with standardised extract of *C. asiatica* (Centell-S) resulted in an increase of the level of bioactive triterpenoids in the systemic circulation. The metabolomic study revealed the correlation between increased bioactive triterpenoids and biomarkers levels that related to neurodegenerative diseases. These results could be beneficial for the further drug development program of Centell-S and piperine combination for clinical neurodegenerative applications.

## Materials and methods

### Chemicals

The Centell-S (purity ≥ 86.7%), piperine (purity ≥ 98.0%) and curcumin (purity ≥ 95.0%) used in the pharmacokinetic experiment were provided by Siam Herbal Innovation Co. Ltd. Analytical grade madecassoside (purity ≥ 96.7%) and madecassic acid (purity ≥ 97.5%) were purchased from Chromadex Corp. Analytical standards of asiaticoside (purity ≥ 98.5%) and asiatic acid (purity ≥ 97.0%) were purchased from Sigma-Aldrich Inc. The internal standards glycyrrhizin (purity ≥ 90.0%) and glycyrrhetinic acid (purity ≥ 98.0%) were purchased from Wako Pure Chemical Industries Ltd.

### Animals

The proposed animal experiment was approved by the Institutional Animal Care and Use Committee of the Chulalongkorn University Laboratory Animal Centre (CULAC), Chulalongkorn University, Bangkok, Thailand (Protocol number 2073029, Approval date: February 5, 2021). For the pharmacokinetic studies, 1.5-year-old male beagle dogs were received from CULAC, Chulalongkorn University and kept in an animal facility for 1 week to acclimatise at 22 ± 2 °C, 50 ± 20% relative humidity, under a 12 h dark/light cycle. The dogs were fed with a normal dog diet (Beauty Pro, Nippon Pet Food Co. Ltd.) once a day and water ad libitum. Two groups of four dogs were randomly used with a 2-week washout period. The dogs were fasted overnight prior to the start of the experiment and up to 4 h post‐administration of the test compounds, but water was freely accessible. After receiving the test substances, the dogs were transferred to a metabolic cage for 48 h for the collection of urine and faeces. All animal procedures were performed in accordance with the ethical principles, regulations, and guidelines for the use of animals (National Research Council of Thailand, 2015). This study was conducted in compliance with the ARRIVE guidelines.

### Pharmacokinetic study

Four experimental groups received the oral test compound once daily for seven consecutive days, as follows: (i) Centell-S 20 mg/kg; (ii) Centell-S 20 mg/kg + piperine 20 mg/kg; (iii) Centell-S 20 mg/kg + curcumin 20 mg/kg; and (iv) Centell-S 20 mg/kg + piperine 20 mg/kg + curcumin 20 mg/kg. The test substance powders received from the suppliers were packed into gelatine capsule, and the dogs were orally administered two to four capsules in the morning, according to the number of test substances required in the experimental plan. Blood samples (2–3 mL) were withdrawn from the cephalic or saphenous vein using an IV catheter and placed in heparinised tubes. Blood collection was conducted on day 1 and day 7 at the following predetermined time intervals: pre-dose, 0.25, 0.5, 1, 2, 4, 8 and 24 h post-dose. The pre-dose and 24 h post-dose blood chemistry parameters were determined by the Small Animal Hospital, Faculty of Veterinary Science, Chulalongkorn University for all dogs, in order to monitor the health status of the dogs.

### Sample preparation for the pharmacokinetic study

Blood samples collected from the dogs were centrifuged at 1500*g* for 10 min at 4 °C for plasma collection. Urine and faeces samples were collected from the metabolic cages for 0–24 h and 24–48 h after administration of the test substances, and faeces samples were homogenised by mixing with methanol and centrifuged at 1500*g* for 10 min at 4 °C to collect the supernatant. All biological samples were kept at − 80 °C until required for analysis. Where the concentration of the samples exceeded the linear calibration curve, blank matrices were used to dilute the sample before protein precipitation. Methanol was used to extract the major triterpenoids from the biological samples for LCMS analysis. In brief, 50 µL of the biological sample was added to 200 µL methanol containing glycyrrhizin and glycyrrhetinic acid as internal standards. The mixture was then centrifuged at 12,000*g* for 10 min at 4 °C to collect the supernatant, and 10 µL of the supernatant was injected into the LCMS system to measure the concentration of the targeted analytes. Method validation was based on our previous report and showed good specificity and linearity for all of the targeted analytes^39^.

### Instrumentation for the pharmacokinetic study

The LCMS system was set up in accordance with the published method, using a Nexera Ultra High-Performance Liquid Chromatography equipped with 8060 triple quadrupole mass spectrometer controlled by LabSolution software version 5.86 (Shimadzu Corp.)^39^. A Synergi Fusion-RP C18 column (Phenomenex Inc.) was selected as the stationary phase and maintained at 40 °C in the column oven. Absolute methanol and 0.2% formic acid in water were used as a mobile phase and were run using a gradient system, as follows: 10% methanol from 0.0 to 0.5 min, increased to 90% methanol from 0.5 to 1.5 min, maintained at 90% methanol from 1.5 to 2.5 min, decreased to 10% methanol from 2.5 to 3.0 min and maintained at 10% methanol until 5.0 min, with a flow rate of 0.5 mL/min. Multiple reaction monitoring was operated in negative mode for madecassoside, asiaticoside, madecassic acid, asiatic acid, glycyrrhizin and glycyrrhetinic acid, with a mass-to-charge ratio of 937.40/503.30, 957.40/469.20, 503.25/437.15, 487.30/409.45, 821.25/350.90 and 469.35/409.40, respectively. The calibration curves of the targeted analytes exhibited good linearity (R^2^ > 0.99), with a concentration range from 1 to 10,000 µg/L for madecassoside and asiaticoside and from 10 to 5000 µg/L for madecassic acid and asiatic acid. The analysis method exhibited good specificity because there had been no observation of any other impurity interferences from the matrix at the retention time of the target analytes and the percentage recovery was over 80% for all of the compounds (Supplementary Figs. S1, S2, and Table S1). The lower limit of quantification (LLOQ) of each of the compounds in this study ranged from 1.00 to 10.00 µg/L, according to the compound.

### High‑resolution accurate‑mass (HRAM) data analysis for the untargeted metabolomic study

Initially, 200 µL of the dog plasma samples at pre-dose and post-dose 4 h were extracted using 400 µL extraction solvent (methanol:chloroform, 3:1). The samples were then vortex-mixed, incubated at 4 °C for 30 min and centrifuged at 13,000*g* for 15 min at 4 °C, and 100 µL of the supernatants were then subjected to analysis by LCMS untargeted metabolomics (high‑resolution accurate‑mass (HRAM) platform). The HRAM platform consisted of an ultra-high pressure liquid chromatograph (UHPLC, Thermo Scientific) coupled to an orbitrap-based mass spectrometer (Orbitrap Exploris480, Thermo Scientific). The mass spectrometer was calibrated for the detection of positive and negative ions on a daily basis, before starting analytical processing, using an ion calibration solution (Pierce FlexMix Calibration Solution, Thermo Scientific). The UHPLC injection sequence consisted of solvent blanks, pooled quality control (QC) samples that were injected before and between plasma sample groups, which included six technical replicates of a pool of aliquots derived from the study plasma samples, and study plasma samples.

Separation of the plasma metabolites was performed using an Accucore-C18 column (Thermo Scientific) maintained at 50 °C. Mobile phase A was 0.1% formic acid in water, while mobile phase B was 0.1% formic acid in methanol. The sample injection volume was 10 µL, and the mobile phase flow rate was 0.6 mL/min for 60 min (0–8 min with 1% mobile phase B, 40–48 min with 99% mobile phase B, and 48.1–60 min with 1% mobile phase B). An orbitrap Exploris 480 mass spectrometry was used with heated electrospray ionization in positive and negative detection mode at a resolution of 120,000 for ddMS^2^. Internal Mass Calibration (EASY-IC, Thermo Scientific) was used for verification of the mass accuracy during the mass detection process. The spray voltage was static as 3500 for positive mode and 2800 for negative mode, and the gas was set as static by sheath gas 50, Aux gas 12 and Sweep gas 2. The ion transfer tube temperature was 263 °C and the vaporizer temperature was 425 °C.

The raw data files acquired from the Xcalibur software (Version 4.4, ThermoFisher Scientific) were primarily processed using Compound Discoverer software (version 3.2.0.421, ThermoFisher Scientific) for the determination and identification of positive polarity and negative polarity metabolites between samples with an untargeted metabolomics workflow. The workflow used the adaptative curve model with 1 min maximum shift and 10 ppm mass tolerance for retention time alignment. The detection compound node required 10 ppm mass tolerance, 30% intensity tolerance for extracted ion chromatograms with a minimum peak intensity of 1,000,000 and 3 signal/noise (S/N) threshold.

All detected compounds were grouped across samples, with 5 ppm mass error and 0.2 min retention time shift by fragment data selection as [M + H] + 1, [M − H] − 1. Missing peaks (not detected initially) in all samples were determined using the fill gaps node algorithm, with 5 ppm mass error and 1.5 S/N threshold for centroid filtering, and QC-based area correction was applied for instrument drift using the cubic spline regression model. Each compound had to be detected in at least 80% of the QC coverage, with maximum QC area relative standard deviation (RSD) at 30% and a maximum corrected QC area RSD of 20%. Compound identification was performed using mzCloud (ddMS2) and ChemSpider against the Human Metabolome database, KEGG, LipidMAPS and NIST (formula or exact mass with 3 ppm mass tolerance). The mzCloud and mzLogic algorithms were applied for similarity searching for all compounds with ddMS2 data, to rank the order of the ChemSpider results. The data were mapped to the Metabolika pathways node and KEGG pathways node by formula or mass, with a mass tolerance of 5 ppm. The pre-processed data were exported to .xlsx files for further statistical analysis.

### Data analysis

After oral administration, pharmacokinetic parameters were determined by PK solution software version 2.0 (Submit Research Services, USA.) using non-compartmental analysis. The maximum plasma concentration (C_max_) and time to reach maximum plasma concentration (T_max_) were calculated directly from the concentration–time curves. The area under the curve from time 0–24 h (AUC_0–24_) was determined by the trapezoidal method and extrapolated to time infinity by the equation AUC_0–inf_ = AUC_0–24_ + (C_last_/k_el_), where C_last_ is the last measurable plasma concentration and k_el_ is the elimination rate constant. The rate of bioavailability was equal to − 2.303 × slope. The mean residence time (MRT) was calculated as AUMC_0–inf_/AUC_0–inf_, where AUMC_0–inf_ is the area under the first moment curve. The volume of distribution (Vd) was equivalent to dose/C_0_, where C_0_ is the concentration of drug measured in plasma at time zero. The total clearance (CL) was equal to dose/AUC_0–inf_, and the elimination half-life was determined by 0.693/k_el_. The accumulation ratio (R_ac_) was calculated as R_ac_ = AUC_0–24, ss_/AUC_0–24, 1_, where AUC_0–24, ss_ is the steady-state areas under the plasma concentration–time curves during a dosing interval (0–24) and AUC_0–24, 1_ represents this parameter after the first dose. The difference in blood chemistry parameters pre-dose and post-dose was compared using Student’s *t* test. SPSS version 22.0 (IBM Corp.) was used to determine statistically significant differences between the experimental groups. The normal distribution of the data was checked using the Shapiro–Wilk test, and a paired Student’s *t* test or Wilcoxon signed-rank test was then performed, where appropriate. The percentage recovery of the test substances was calculated by dividing the total amount of test substance in the urine or faeces by the delivered dose. All data is presented as the mean ± standard deviation, except for T_max_ and half-life, which are shown as median (IQR). A *p*-value < 0.05 is considered statistically significant.

## Supplementary Information

Supplementary Information.

## Data Availability

The data that support the findings of this study are available from the corresponding author upon reasonable request. Some data may not be available because of privacy or ethical restrictions.

## Abbreviations

ALT: Alanine aminotransferase
ASA: Asiatic acid
ASS: Asiaticoside
AST: Aspartate aminotransferase
AUC: Area under the curve
CL: Total clearance
C_max_: Maximum plasma concentration
Centell-S: Standardised extract of *Centella asiatica* with improved water solubility
Cur: Curcumin
HRAM: High resolution accurate mass
LCMS: Liquid chromatography tandem mass spectrometry
LLOQ: Lower limit of quantification
MDA: Madecassic acid
MDS: Madecassoside
MRT: Mean residence time
PCA: Principle component analysis
Pip: Piperine
QC: Quality control
RSD: Relative standard deviation
S/N: Signal to noise ratio
T_max_: Time to reach maximum plasma concentration
Vd: Volume of distribution
