# Supplementary material for: Bioenhancing effects of piperine and curcumin on triterpenoid pharmacokinetics and neurodegenerative metabolomes from Centella asiatica extract in beagle dogs
Source: Sci Rep. 2022 Dec 1;12:20789. doi: 10.1038/s41598-022-24935-7 (PMC9715946; doi:10.1038/s41598-022-24935-7)
Supplement: Supplementary file 1 — Supplementary Information. [file 41598_2022_24935_MOESM1_ESM.docx]

Glycyrrhetinic acid

Glycyrrhizin

Asiatic acid

Madecassic acid

Asiaticoside

Madecassoside

**Figure S1.** Chromatogram and retention time of target analytes in solvent.

Glycyrrhetinic acid

Glycyrrhizin

Asiatic acid

Madecassic acid

Asiaticoside

Madecassoside

**Figure S2.** Chromatogram and retention time of target analytes in dog plasma.

**Table S1.** Bioanalytical method validation of the target analytes using LC-MS/MS.

|  | Conditions | Concentration (µg/L) | Mean | SD | Accuracy (%) | Precision (%CV) | Recovery (%) |
| --- | --- | --- | --- | --- | --- | --- | --- |
| Madecassoside | Intraday | 1000  300  10 | 1117.32  312.76  11.40 | 21.70  11.69  1.39 | 11.73  4.25  14.00 | 1.94  3.74  12.16 | 90.34  85.58  92.46 |
|  | Interday | 1000  300  10 | 1132.26  316.56  10.48 | 21.75  10.31  0.73 | 13.23  5.52  4.77 | 0.24  3.26  7.00 | 87.91  84.69  90.32 |
|  | Storage at 4°C | 1000  300  10 | 1016.04  308.28  10.23 | 23.65  11.44  0.27 | 1.60  2.76  2.25 | 2.33  3.71  2.60 | 85.11  90.80  89.41 |
|  | 3 freeze-thaw cycles | 1000  300  10 | 963.83  328.23  9.97 | 23.69  6.42  0.34 | -3.62  9.41  -0.27 | 2.46  1.96  3.36 | 87.35  89.48  92.99 |
|  | Storage at -20°C | 1000  300  10 | 1147.30  332.76  11.16 | 0.06  6.93  0.48 | 14.73  10.92  11.60 | 0.01  2.08  4.33 | 85.44  92.01  93.95 |
|  | Autosampler | 1000  300  10 | 1111.14  325.04  10.55 | 47.46  7.34  0.03 | 11.11  8.35  5.50 | 4.27  2.26  0.33 | 88.03  90.58  91.13 |
| Asiaticoside | Intraday | 1000  300  10 | 1126.51  314.26  10.19 | 89.28  4.74  0.63 | 12.65  4.75  1.94 | 7.93  1.51  6.16 | 88.40  90.12  91.35 |
|  | Interday | 1000  300  10 | 1104.44  320.32  11.43 | 58.42  10.89  0.21 | 10.44  6.77  1.82 | 5.29  3.40  1.82 | 87.59  89.23  90.62 |
|  | Storage at 4°C | 1000  300  10 | 1121.00  304.68  10.75 | 35.59  6.21  1.20 | 12.10  1.56  7.55 | 3.18  2.04  11.18 | 84.89  86.95  93.55 |
|  | 3 freeze-thaw cycles | 1000  300  10 | 1041.35  329.58  11.11 | 78.54  13.36  0.27 | 4.14  9.86  11.07 | 7.54  4.05  2.44 | 82.34  87.31  89.09 |
|  | Storage at -20°C | 1000  300  10 | 1078.48  309.95  10.57 | 24.88  0.03  0.14 | 7.85  3.32  5.71 | 2.31  3.32  5.71 | 85.43  89.88  92.08 |
|  | Autosampler | 1000  300  10 | 1101.69  333.14  11.28 | 57.96  0.33  0.98 | 10.17  11.05  12.77 | 5.26  0.10  8.73 | 81.08  84.04  91.58 |
| Madecassic acid | Intraday | 1000  300  10 | 1046.27  322.51  10.96 | 69.73  14.82  1.13 | 4.63  7.50  9.57 | 6.66  4.59  10.35 | 87.90  85.61  90.64 |
|  | Interday | 1000  300  10 | 1159.68  327.36  10.59 | 36.30  23.42  0.06 | 13.97  9.12  5.87 | 3.13  7.15  0.52 | 81.97  90.45  87.93 |
|  | Storage at 4°C | 1000  300  10 | 1037.66  299.47  10.40 | 129.83  10.40  0.34 | 3.77  -0.18  4.00 | 12.51  3.47  3.23 | 88.71  85.93  93.87 |
|  | 3 freeze-thaw cycles | 1000  300  10 | 1125.45  302.05  10.55 | 31.99  5.61  0.21 | 12.55  0.68  5.53 | 0.18  1.86  1.97 | 89.37  88.75  90.04 |
|  | Storage at -20°C | 1000  300  10 | 1098.75  314.56  10.28 | 86.94  7.01  0.06 | 9.87  4.85  2.80 | 7.91  2.23  0.61 | 83.84  87.66  89.09 |
|  | Autosampler | 1000  300  10 | 1041.72  314.90  9.70 | 37.63  21.99  0.42 | 4.17  4.97  -3.00 | 3.61  6.98  4.35 | 84.35  90.03  91.96 |
| Asiatic acid | Intraday | 1000  300  10 | 1041.55  300.21  9.62 | 101.97  10.30  0.94 | 4.15  0.07  -3.83 | 9.79  3.43  9.77 | 88.72  86.08  93.04 |
|  | Interday | 1000  300  10 | 1078.92  332.05  10.69 | 49.96  8.06  0.68 | 7.89  10.68  6.91 | 4.63  2.43  6.38 | 83.66  87.04  91.54 |
|  | Storage at 4°C | 1000  300  10 | 1103.33  308.18  9.79 | 39.25  14.15  0.79 | 10.33  2.73  -2.07 | 3.56  4.59  8.11 | 84.88  87.01  89.86 |
|  | 3 freeze-thaw cycles | 1000  300  10 | 1103.96  325.87  11.22 | 66.66  5.04  0.61 | 10.40  8.62  12.17 | 6.04  1.55  5.46 | 88.39  85.53  93.88 |
|  | Storage at -20°C | 1000  300  10 | 1083.01  313.62  10.29 | 7.80  3.05  0.89 | 8.30  4.54  2.90 | 0.72  0.97  8.63 | 82.89  85.92  87.97 |
|  | Autosampler | 1000  300  10 | 1094.56  328.73  9.66 | 47.48  10.33  0.98 | 9.46  9.58  -3.43 | 4.34  3.14  10.12 | 84.97  88.59  92.06 |
